# A Novel Digestive α-Amylase from Blue Crab (*Portunus* *segnis*) Viscera: Purification, Biochemical Characterization and Application for the Improvement of Antioxidant Potential of Oat Flour

**DOI:** 10.3390/ijms22031070

**Published:** 2021-01-22

**Authors:** Hana Maalej, Amina Maalej, Sawsan Affes, Noomen Hmidet, Moncef Nasri

**Affiliations:** 1 Laboratory of Enzyme Engineering and Microbiology, National School of Engineering of Sfax (ENIS), University of Sfax, P.O. Box 1173, Sfax 3038, Tunisia; sawsanaffes170@gmail.com (S.A.); hmidet_noomen@yahoo.fr (N.H.); mon_nas@yahoo.fr (M.N.); 2 Department of Life Sciences, Faculty of Science of Gabes, Omar Ibn Khattab Street, Gabes 6029, Tunisia; 3 Environmental Bioprocesses Laboratory, Sfax Biotechnology Center, P.O. Box 1177, Sfax 3038, Tunisia; maalejamina@yahoo.fr

**Keywords:** blue crab, *Portunus segnis*, digestive α-amylase, purification, biochemical characterization, antioxidant potential, oat flour, food industries

## Abstract

This study reports on the purification and characterization of a digestive α-amylase from blue crab (*Portunus* *segnis*) viscera designated Blue Crab Amylase (BCA). The enzyme was purified to homogeneity by ultrafiltration, Sephadex G-100 gel filtration and Sepharose mono Q anion exchange chromatography, with the final purification fold of 424.02, specific activity of 1390.8 U mg^−1^ and 27.8% recovery. BCA, showing a molecular weight of approximately 45 kDa, possesses desirable biotechnological features, such as optimal temperature of 50 °C, interesting thermal stability which is enhanced in the presence of starch, high stability towards surfactants (Tween 20, Tween 80 and Triton X-100), high specific activity, quite high storage and broad pH range stability. The enzyme displayed *Km* and *Vmax* values, of 7.5 ± 0.25 mg mL^−1^ and 2000 ± 23 μmol min^−1^ mg^−1^ for potato starch, respectively. It hydrolyzed various carbohydrates and produced maltose, maltotriose and maltotetraose as the major end products of starch hydrolysis. In addition, the purified enzyme was successfully utilized for the improvement of the antioxidant potential of oat flour, which could be extended to other cereals. Interestingly, besides its suitability for application in different industrial sectors, especially food industries, the biochemical properties of BCA from the blue crab viscera provide novel features with other marine-derived enzymes and better understanding of the biodegradability of carbohydrates in marine environments, particularly in invasive alien crustaceans.

## 1. Introduction

Owing to its vast diversity of organisms and habitats, the marine world is considered a prominent potential source of unique and valuable bioactive compounds, particularly of carbohydrate-active enzymes [1]. As compared to their terrestrial counterparts, marine-derived enzymes are valuable as food ingredients due to their specificity, diverse properties, salt tolerance, as well as their ability to function at extremes of pH and temperature [2,3,4]. 

On the other hand, the activities related to the fishing sector generate substantial quantities of byproducts produced as a result of fish and shellfish processing, such as heads, skin, bones, exoskeletons, shells and viscera, which are often discarded as industrial waste and thus cause numerous environmental problems [5]. Interestingly, recovery of digestive enzymes from such byproducts is a very exciting and promising alternative [6]. In fact, fish viscera have been reported to be a good source of digestive enzymes with interesting properties that are highly valued in a wide range of industrial applications [7,8,9]. Besides proteases, α-amylases (1,4-α-D-glucan glucanohydrolase, EC 3.2.1.1), which represent about 30% of the whole enzyme market in the world, are endo-acting enzymes belonging to glycoside hydrolase family 13 (GH13) [10,11,12,13,14]. Such enzymes catalyze the hydrolysis, in a random fashion, of α-1,4-glycosidic bonds in amylose, amylopectin and starch-related polysaccharides thus yielding low molecular weight saccharides, including maltodextrins, maltose and glucose molecules. α-Amylases are known as commercially relevant enzymes for numerous important industrial applications in food (starch liquefaction and saccharification, baking industries, preparation of digestive aids, etc.), textile, detergents, paper, brewing, distilling and pharmaceutical industries [15,16]. Hence, for such industrial purposes, considerable attention has been focused on the search for low-cost sources for obtaining new α-amylases with improved/novel properties [17,18]. 

Several α-amylases have been identified in marine bacteria, including *Pseudoalteromonas* sp. [19], *Carnobacterium* and *Psychrobacter* strains [20], *Aeromonas salmonicida* ssp. *salmonicida* A449 [21], *Catenovulum* sp. X3 [22] and *Zunongwangia profunda* [23].

Recently recorded at the southern Tunisian coasts, particularly in the Gulf of Gabes, the blue crab *Portunus segnis* is described as a voracious alien species having negative ecological and socioeconomic impacts, thus representing a major threat to marine biodiversity by displacing native species, changing community structure and food webs [24]. As a consequence, the government has allocated funds to boost valorization of such invasive crustaceans in an attempt to turn the blue crab commercial fishery profitable [25]. In spite of its abundance and to the best of our knowledge, until now, no information has been reported on the preparation of visceral extracts with amylase activity, especially from blue crab viscera. 

Based on these considerations, this study focuses on a novel digestive α-amylase extraction and purification from the blue crab (*Portunus segnis)* viscera, as well as its biochemical characterization and application for the improvement of antioxidant potential of oat flour. The research aims to recover promising added value compounds from blue crab byproducts to help solve the problem of disposal and create extra revenue as well as to provide fundamental information needed for commercial application of this enzyme in the food industry.

## 2. Results

### 2.1. Purification of the BCA α-Amylase

**Figure 1:**
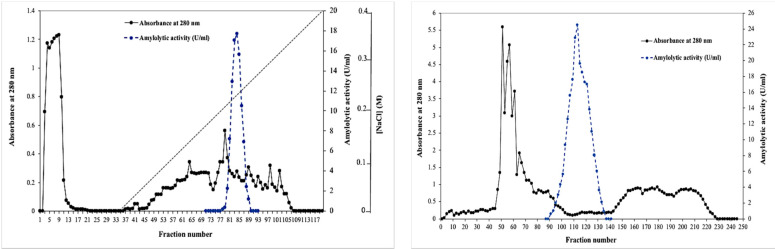
Elution profiles of BCA on Sephadex G-100 (**a**) and Mono Q Sepharose (**b**) column chromatography.

In order to establish the specific properties of the digestive α-amylase from *Portunus segnis* (BCA), the crude amylolytic extract of the blue crab visceral waste has been subjected to a series of treatments, each aiming at the subsequent improvement of the purity level. The BCA enzyme was purified by a three-step procedure as described in the Section 3. Firstly, the crude enzyme was concentrated by using an ultrafiltration cell equipped with a cut-off membrane of 10 kDa, enabling it to reach a specific activity 4.71 times higher than that of the crude extract with 85% recovery. Then, the concentrated enzyme was loaded on a Sephadex G-100 gel filtration column (Sigma-Aldrich, Tunisia) previously equilibrated with 25 mM Tris–HCl buffer (buffer A, pH 8.0) containing 0.5‰ Triton X-100 to remove most of the contaminating proteins, resulting in a substantial increase in the purification fold of 31.12 with a yield of 49%. The elution profile showed a single peak of amylolytic activity (Figure 1a). Finally, active fractions from this peak were collected and subjected to a Sepharose Mono Q anion exchange chromatography already equilibrated and washed with buffer A. Unadsorbed proteins were then eluted with the same buffer. Binding proteins were eluted with a linear gradient of NaCl 0–0.4 M in the same buffer at a flow rate of 1.25 mL min^−1^. The Mono Q Sepharose profile represented in Figure 1b indicated that the BCA α-amylase was eluted at 220–260 mM NaCl (fractions 78–90). All the results of the purification procedure are summarized in Table 1. The final purification step allowed achieving a specific activity of 1390.8 U mg^−1^ and 424.02-fold purification with 27.8% yield. 

The degree of purity needed and number of purification steps involved depend on the purpose for which the enzyme is required. In the present study, in an attempt to assess its biochemical characterization, α-amylase was highly purified by three sequential steps, including ultrafiltration, Sephadex G-100 gel filtration and Mono Q Sepharose ion-exchange chromatography. Tsao et al. [26] reported 1076.3- and 1812.9-fold purification of two digestive amylases AI-1 and AI-2, respectively, from the viscera of hard clam *Meretrix lusoria* using a four-step purification procedure. However, an 18-fold purification was achieved for amylase AII purified by six steps from the same species with a yield of only 1.2%. Hsieh et al. [27] purified an amylase from small abalone *Haliotis sieboldii* viscera in three steps, achieving 853.2 U mg^−1^ of specific amylase activity, 161-fold purification and 13% of recovery. An α-amylase was purified 369-fold from the crystalline short-necked clam *Ruditapes philippinarum* by a combination of gel filtration on Sephadex G-50, Bio-Gel P-10 and Sephadex G-25, with a recovery of 21% [28]. Interestingly, using the present purification process, higher enzyme purification fold (424.02) and yield (27.8%) were achieved with fewer purification steps. Such purification procedure can be easily adopted for large-scale purification since it is simple and straightforward.

**Figure 2:**
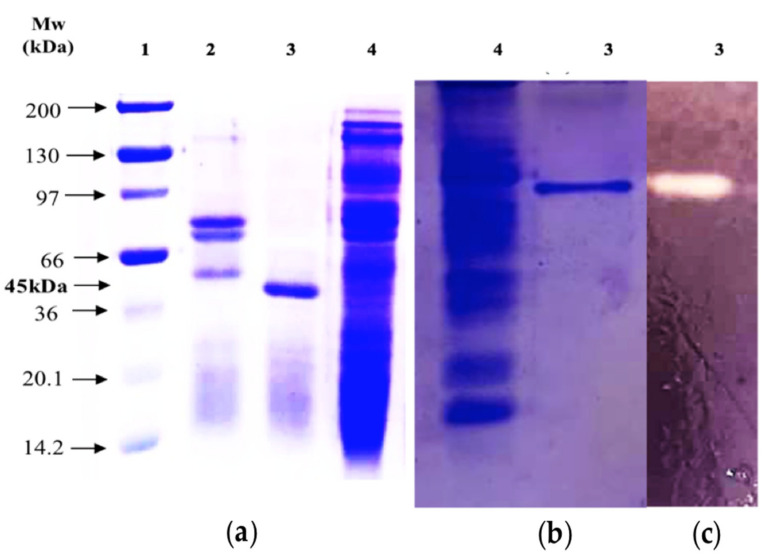
SDS-PAGE (**a**), Native PAGE (**b**) and zymogram detection of amylolytic activity (**c**) of the purified BCA from the blue crab viscera. Lane 1, standard proteins marker; lane 2, G-100 purified; lane 3, Mono Q-purified BCA; and lane 4, visceral extract.

Then, to analyze the homogeneity and molecular mass of the purified BCA, SDS-PAGE and Native PAGE were performed. As shown by SDS-PAGE, the molecular weight of the purified amylolytic enzyme was estimated to be approximately 45 kDa (Figure 2a). Additionally, this preparation was homogeneous as it exhibited a single band of proteins on both SDS-PAGE and Native PAGE (Figure 2b), thus indicating its high purity degree. Further, the zymogram activity staining also revealed only one zone of amylolytic activity for the purified BCA (Figure 2c). The molecular weight of the purified BCA was comparable with previously reported marine amylases, such as α-amylase (49.3 kDa) from *Alteromonas haloplanctis* [29], digestive amylase AI-1 (49.6 kDa) from hard clam *Meretrix lusoria* [26] and α-amylase (46 kDa) from the copepod *Acartia clausi* [30]. At the same time, it was smaller than the small abalone amylase II-1 (55.7 kDa) [31], the tilapia intestinal amylase (56.4 kDa) [32], the amylase (59 kDa) from small abalone *Haliotis sieboldii* viscera [27] and the digestive amylases AI-2 and AII (58.7 and 100 kDa, respectively) from hard clam *Meretrix lusoria* [26]. On the other hand, low molecular mass amylase (28 kDa) produced by a bacterium isolated from the distal intestine of a freshwater fish, *Anabas testudineus*, has also been reported in the literature [33].

### 2.2. Biochemical Characterization of the Purified BCA

#### 2.2.1. Effects of Temperature on Enzyme Activity and Stability

**Figure 3:**
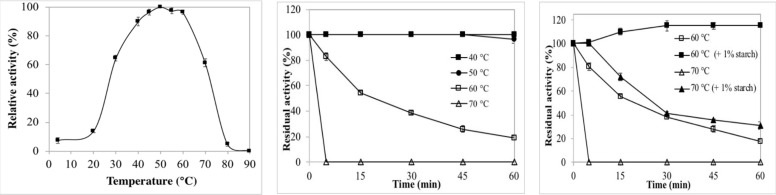
Temperature profile (**a**) and thermal stability of the purified BCA in the absence (**b**) and presence (**c**) of starch. Amylolytic activity was assayed at different temperatures ranging from 4 to 90 °C at pH 8.0. The activity of the enzyme at 50 °C was taken as 100%. To assess the thermostability of BCA, the enzyme was heated at the indicated temperatures. The effect of starch on the enzyme’s thermostability enhancement was also assessed. The residual activity was assayed at pH 8.0 and 50 °C. The non-heated enzyme was taken as the control (100%).

The temperature profile of the digestive BCA is shown in Figure 3a. The purified α-amylase was found to be active at the temperature range from 30 to 70 °C retaining more than 50% of its maximum activity shown at 50 °C. The enzyme activity decreased sharply at temperatures above 70 °C where approximately 95% loss in the enzyme activity was detected at 80 °C. The optimal temperatures for α-amylases from short-necked clam (36 °C) [28], *Penaeus japonicus* (40 °C) [34], copepod (40 °C) [30], tilapia intestine (40 °C) [35], *Perna viridis* digestive gland (43 °C) [36] and amylase AI-1 (40 °C) from hard clam *Meretrix lusoria* viscera [26] were lower than those for the purified BCA. The optimal temperature of the purified α-amylase from blue crab viscera was similar to those of amylases AI-2 and AII purified from hard clam *Meretrix lusoria* viscera [26] and amylases II-1 and II-2 from the small abalone [31]. Hmidet et al. [37] reported the optimal temperatures for amylolytic activity to be 40 °C for *Sardinella* and grey triggerfish visceral amylases and 50 °C for the barbel visceral amylase. AmyZ1, an α-amylase from marine bacterium *Pontibacillus* sp. ZY, showed the highest activity at 35 °C and maintained more than 40% of its optimal activity at 20 °C, indicating the “cold-active” catalytic ability [38].

Besides optimal temperature, the enzyme’s thermal stability is considered an important feature making its use possible in numerous biotechnological processes that require a wide range of temperatures [39]. The time course of BCA thermal inactivation was followed at temperatures ranging from 30 to 70 °C (Figure 3b). The thermal stability pattern showed a highly stable enzyme at temperatures below 50 °C retaining its full activity after 60 min. Its original activity remained at 96% after incubation at 50 °C for 60 min. The enzyme lost approximately 45, 62 and 82% activity within 15, 30 and 60 min of incubation at 60 °C, respectively. After only 5 min of incubation at 70 °C, the purified enzyme was completely inactivated. According to the literature, most marine-derived α-amylases have been reported to be relatively stable at lower temperatures, as compared to those from other origins [23,40,41]. Fang et al. [38] reported that AmyZ1, a marine-derived α-amylase, exhibited poor thermostability with about 60% of the activity lost within 10 min at 35 °C. In comparison, the introduction of Ca^2+^ ions significantly improved the AmyZ1 catalytic stability. The purified amylases AI-1, AI-2 and AII from the viscera of hard clam *Meretrix lusoria* were very stable at temperatures <35 °C, <40 °C and <40 °C, respectively. At the same time, they retained less than 20% of their initial activity once incubated at 55 °C for 30 min [26].

Interestingly, as shown in Figure 3c, the addition of starch (1%) led to an improvement in BCA thermal stability at both of the temperatures (60 and 70 °C). Indeed, after pre-incubation for 60 min at 60 °C, the purified enzyme retained only 17.72% of its activity; however, its full activity was restored in the presence of 1% starch under the same treatment. Moreover, due to the presence of its substrate, the enzyme’s residual activity increased from 0 to 100, 41.5 and 31% after 5, 30 and 60 min of incubation at 70 °C, respectively. Hence, starch may be considered an interesting stabilizer of BCA activity against thermal treatment. Thermostability enhancement of purified α-amylases in the presence of its substrate was supported by the results of Maalej et al. [42]. Furthermore, Koyama et al. [43] reported that low concentrations of starch enhanced thermostability of the human salivary α-amylase (HSA) which binds starch, forming an enzyme–substrate complex that stabilizes the enzyme. 

#### 2.2.2. Effects of pH on Enzyme Activity and Stability

**Figure 4:**
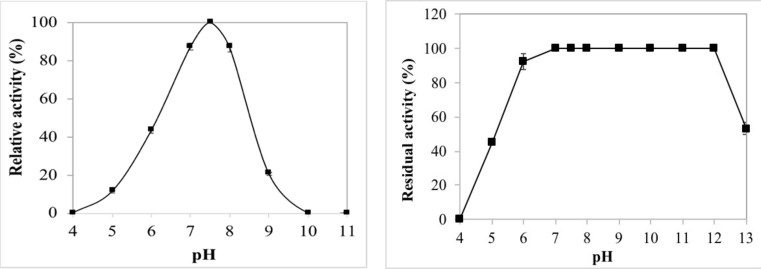
pH profile (**a**) and pH stability (**b**) of the purified BCA. Amylolytic activity was tested at 50 °C at the pH values ranging from 3.0 to 13.0. The maximum activity obtained at pH 7.5 was considered as 100%. To assess the pH stability, BCA was pre-incubated at the indicated pH at 30 °C for 60 min and its residual activity was determined at pH 7.5 and 50 °C. The activity of the enzyme before incubation was taken as 100%. Buffer solutions used for pH activity and stability are presented in Section 3.

The pH activity profile of the purified blue crab visceral α-amylase was investigated by using potato starch as a substrate over the pH range of 3.0–13.0 at 50 °C, as shown in Figure 4a. BCA exhibited the maximal activity at pH 7.5. The activity of the enzyme was high in the pH range of 7.0–8.0, but considerable loss of activity was observed at acidic and alkaline pH values. No activity was found at pH 4.0 and 10.0. Loss of enzymatic activity in more extreme ranges of pH may be caused by electrostatic repulsion deriving from the high net charge in the protein molecules [44]. The optimal pH of purified BCA was close to those of hard clam *Meretrix lusoria* visceral amylases AI-1 (pH 6.5), AI-2 (pH 7.5) and AII (pH 6.8) [26]. Hmidet et al. [37] reported pH 8.0 as optimal for activity of α-amylases from visceral extracts of barbel and grey triggerfish. While they found that the activity of the *Sardinella* viscera showed two optimal pH at 6.0 and 8.0. 

The pH stability profile of BCA, illustrated in Figure 4b, indicated that the purified enzyme was completely stable over a broad pH range (pH 7.0–12.0) after 1 h pre-incubation at 30 °C with more than 90% of the activity recovered at pH 6.0. In this regard, BCA showed greater stability after pre-incubation at neutral or alkaline pH values. Similar behavior was reported by Fernandez et al. [45] for α-amylase activity in five species of Mediterranean sparid fish, showing low stability at acid pH values. The pH stability of the digestive α-amylase BCA was higher than those of AmyZ1 which exhibited poor pH stability [38], amylase from the small abalone *Haliotis sieboldii* viscera (pH 6.0–8.0) [27] and tilapia intestinal amylase (pH 5.5–7.5) [32].

#### 2.2.3. Effects of Metal Ions on BCA Activity

The effects of various metal chloride ions (5 mM) on enzyme activity were determined at 50 °C and pH 7.5. As shown in Table 2, only 2 ions (Hg^2+^ and Zn^2+^) seemed to have a total inhibitory effect, a strong decrease in activity was observed in the presence of Mn^2+^, Cu^2+^, Fe^2+^ and Co^2+^, as compared to the control. A low decrease in BCA activity was detected in the presence of Al^2+^ (91.3 ± 0.98%), K^+^ (95.5 ± 0.61%) and Ca^2+^ (97 ± 1.4%). At the same time the enzyme was activated in the presence of Mg^2+^ and Ba^2+^ ions by 109.7 ± 0.67% and 118.5 ± 2.12%, respectively. The same stimulatory effect of Mg^2+^ and Ba^2+^ was also reported for the amylase from the small abalone *Haliotis sieboldii* [27]. Mg^2+^ also activates short-necked clam [28], tilapia intestinal AA-2 and AB-2 [32], hard clam *Meretrix lusoria* visceral AI-1, AI-2 and AII [26] as well as small abalone II-1 and II-2 [31] amylases. Similarly to the BCA amylase, Hg^2+^ inhibits α-amylases of the tilapia stomach [35], amylases AI-1, AI-2 and AII [26], the short-necked clam amylase [28], small abalone amylases II-1 and II-2 [27] as well as digestive amylases AI-1, AI-2 and AII [26]. In general, amylases are strongly inhibited by Hg^2+^, known to act as a thiol group inhibitor, suggesting the existence of cysteine residue on or close to their active sites. On the other hand, other properties, including the Ca^2+^-binding ability of amylases, were found to be dependent on the enzyme sources [46]. In the present study, the BCA amylase was not activated by Ca^2+^ (97 ± 1.4%), and thus was considered to be a Ca^2+^-independent enzyme. Similarly, Tsao et al. [26] found that AI-1, AI-2 and AII amylases were not enhanced by Ca^2+^, while Hsieh et al. [27] reported a two-fold increase in the small abalone amylase activity (210%) in the presence of 5 mM Ca^2+^.

#### 2.2.4. Effects of Surfactants and Enzyme Inhibitors on BCA Stability

Several surfactants and enzyme inhibitors were assayed for their effects on BCA stability as indicated in Table 3. While BCA revealed moderate stability in the presence of 5% (*v/v*) of non-ionic surfactants, where it could retain approximately more than 71% residual activity, the strong anionic surfactant (SDS) drastically inactivated the purified enzyme where approximately 74% and 83% of its original activity were lost at 1% and 2%, respectively. Aygan et al. [47] reported that SDS, which shows the enzyme proportion of the hydrophobic amino acid composition, was the most effective effector. Chai et al. [48] reported that the α-amylase activity was strongly reduced in the presence of 1% SDS.

Unlike the α-amylase AmyZ1 of the marine bacterium *Pontibacillus* sp. ZY [38], the amylase SA-I from the small abalone *Haliotis sieboldii* [27], hard clam amylases AI-1, AI-2 and AII [26], BCA activity was slightly inhibited (7% of inhibition) by the chelating reagent EDTA, which could lead to the conclusion that the purified α-amylase from blue crab viscera is not a metalloenzyme. Fincan et al. [49] reported the same behavior of the α-amylase from thermophilic *Anoxybacillus flavithermus* marine bacteria towards EDTA. Furthermore, BCA α-amylase appears to be stable (93.9 ± 2.68%) in the presence of β-mercaptoethanol, suggesting therefore that disulfide bonds were not essential to maintain the active conformation of the α-amylase.

### 2.3. Kinetic Study and Thin-Layer Chromatographic (TLC) Analysis

#### 2.3.1. Substrate Specificity Profile

The substrate hydrolysis rate may be affected by the structure of substrate molecules, substrate size and the types of the bonds existing in the substrate chain [50]. Therefore, the purified enzyme was tested for its ability to break down several carbohydrate polymers, including starch (potato, wheat and maize), amylose, amylopectin, α-cyclodextrins, dextrins, carboxymethylcellulose (CMC) and chitosan, at a final concentration of 1% (*w/v*) under the standard assay conditions. As shown in Table 4, the BCA enzyme hydrolyzed soluble starch from potatoes with the highest specificity (100%), followed by wheat starch (96.2 ± 1.05%). Furthermore, maize starch and amylopectin were hydrolyzed with moderate specificity (83.4 ± 1.55% and 71.13 ± 0.74%, respectively) and amylose at a low relative rate of 17.8 ± 2.25%, indicating that it had the capacity to hydrolyze both α-1,4 and α-1,6-glycosidic bonds. No activity was detected on CMC, α-cyclodextrin, dextrin and chitosan substrates, which is consistent with the fact that BCA might be an exo-α-amylase.

#### 2.3.2. Determination of Kinetic Parameters

**Figure 5:**
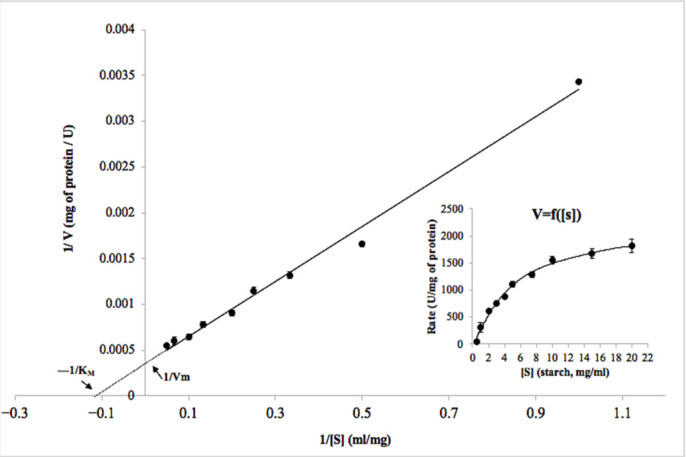
Lineweaver–Burk plot for the BCA α-amylase from blue crab viscera. Starch concentration varied in the range of 0–20 mg mL^−1^. *K_m_* and *V_max_* values were calculated from the plot. Inset shows the V = f ([S]) plot.

From the kinetic study, it seems that the BCA α-amylase displayed the classical Michaelis–Menten kinetics when potato starch was used as substrate (Figure 5). The BCA kinetic parameters *K_m_* and *V_max_* were found to be 7.5 ± 0.25 mg mL^−1^ and 2000 ± 23 μmol min^−1^ mg^−1^, respectively. Fang et al. [38] reported that *K_m_* and *V_max_* of AmyZ1, an α-amylase from a marine bacterium, were 8.85 ± 0.44 mg mL^−1^ and 17,837 ± 440 U mg^−1^ using raw rice starch as the substrate. The marine α-amylase AmyP preferred raw rice starch as the substrate. However, the specific activity of BCA was higher by about 17 times than that of AmyP [51]. Interestingly, BCA showed higher specific activity toward potato starch than that of other amylases derived from bacteria, fungi, and yeast, including the amylase from *Bacillus amyloliquefaciens* [52] and the α-amylase from *Bacillus* sp. YX-1 [53], thus demonstrating important features for its use in biotechnological processes. Only few enzymes exhibited specific activity exceeding 1000 U mg^−1^ for the same substrate, such as AmyZ1 from *Pontibacillus* sp. ZY (2623 U mg^−1^), RoAmy from *Rhizopus oryzae* (1089 U mg^−1^) and TdAmyA from *Thermomyces dupontii* (1315 U mg^−1^) [38].

#### 2.3.3. TLC Analysis of Starch and Maltooligosaccharides Hydrolysis Products

**Figure 6:**
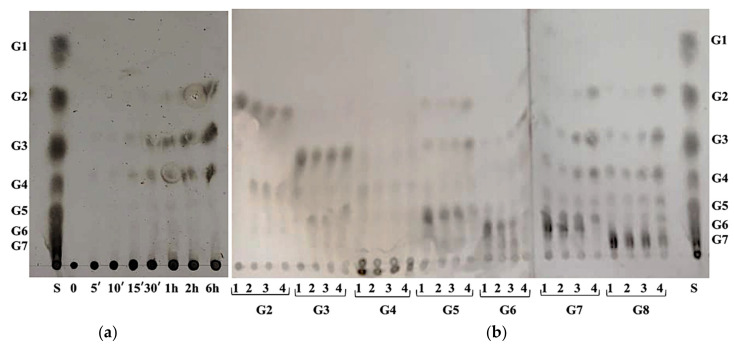
TLC analyses of BCA products from potato starch after 0, 5, 10, 15, 30 min, 1, 2 and 6 h of reaction (**a**) and various maltooligosaccharides (G2, G3, G4, G5, G6, G7 and G8) after 0 (1), 5 (2), 30 (3) and 60 min (4) (**b**). S: maltodextrins (G1 to G7). The reaction mixture contained 0.2 U of α-amylase activity and 1% substrate in 0.1 M Tris–HCl buffer (pH 7.5) was incubated at 50 °C. The hydrolysis was stopped by heating at 100 °C for 5 min. Approximately 5 µL of each hydrolysate were spotted onto the TLC plate.

In order to analyze reaction products, potato starch and various maltooligosaccharides (G2–G8) were hydrolyzed with the purified blue crab digestive α-amylase, and the resulting reaction products were analyzed by TLC using a silica gel plate and a suitable solvent system (Figure 6). 

As shown in Figure 6a, the main hydrolysis products of potato starch (after 6 h of incubation) were maltotetraose, maltotriose and maltose.

To gain more information about the action pattern of the BCA enzyme, intermediate saccharides of starch hydrolysate were used as substrates and end product profiles were analyzed by TLC. As shown in Figure 6b, BCA did not hydrolyze maltose, maltotriose and maltotetraose but was less active on maltopentaose and maltohexaose. The hydrolysis of maltoheptaose leads to G4, G3, G2 and G5, while that of maltooctaose was a mixture of maltooligosaccharides composed of G6, G5, G4, G3 and G2. Therefore, the BCA enzyme could be classified as a liquefying-type α-amylase able to generate maltooligosaccharides in the early time of incubation without glucose production. On the other hand, it is also interesting to note that maltose and maltotriose hydrolysis generated maltooligosaccharides (G4 and G6, respectively) longer than the used substrates, suggesting the presence of a transglycosylation reaction. The predominant end products of raw rice starch after hydrolysis by AmyZ1 were G2, G3 and G5, followed by G1 and G4 [38]. At the same time, the small abalone amylase could hydrolyze starch into glucose and maltose after 12 h of reaction [27]. The hydrolysates of amylose by amylases AI-1, AI-2 and AII from viscera of hard clam *Meretrix lusoria* were found to be glucose/maltose, glucose/maltose and glucose, respectively [31]. Hmidet et al. [37] reported that the main hydrolysis products of potato starch by digestive amylases of barbel (*Barbus callensis*), grey triggerfish (*Balistes capriscus*) and *Sardinella aurita* viscera were maltose and glucose. Interestingly, the amylase from blue crab viscera could be considered a potential candidate for use in the production of syrups rich in maltodextrins and especially free of glucose. Previously reported functional properties make maltodextrins important ingredients in modern food formulation. Indeed, they are able to affect browning reactions, sweetness, depression of freezing points, crystal growth, solubility, hygroscopicity and help to mimic fat to reduce energy [54].

### 2.4. Storage Stability

Since industrial applications almost require a viable enzyme that retains its activity for extended shipping and storage periods, the effect of storage on the purified BCA activity was studied to assess its long-term stability. The enzyme retained about 75% of its initial activity upon storage for 90 days. After one year of storage, the activity dropped to 30%. Such results confirm a quite stable nature of the BCA α-amylase, thus making it suitable for application in various industrial processes.

### 2.5. Application of Purified BCA for the Improvement of Antioxidant Potential of Oat

2,2-Diphenyl-1-picrylhydrazyl (DPPH) radical scavenging activities of untreated (control) and BCA-treated oat flour were investigated, with respect to gallic acid used as the standard antioxidant. Interestingly, an increase of about 1.75 times was observed, as compared to the control (Table 5). On the other hand, oat treatment with the BCA α-amylase increases the soluble phenolic acid content, expressed as the gallic acid equivalent in μmol g^−1^ oat, which was recorded to be 23 ± 2.5 and 75 ± 4.25 in the control and BCA-treated oats, respectively. Fardet et al. [55] reported that phenolic compounds present in cereals possess antioxidant potential. However, since these polyphenols are present as an insoluble bound form linked with the carbohydrate moiety by glycosidic and ester bonds, their bioavailability is hampered, thus lowering their antioxidant potential. Carbohydrate-degrading enzymes hydrolyze carbohydrate moieties, thus increasing the availability of phenols. As reported in the literature, among other commercial enzymes, α-amylases have been used to explore the antioxidant capacity of rice, wheat, oat bran, etc. [56]. 

## 3. Materials and Methods

### 3.1. Obtaining the Raw Materials 

Blue crabs (*Portunus segnis*) were obtained from the coastal marine area of Sfax (Tunisia). The material was refrigerated and taken to the Laboratory of Enzyme Engineering and Microbiology, Sfax, for processing and production of the crude enzyme extract. 

### 3.2. Preparation of Crude Digestive Enzyme Extract

Viscera from blue crabs were separated manually and stored at −20 °C until used for enzyme extraction. For the preparation of the crude amylase, viscera were thawed at room temperature and homogenized with 100 mM Tris-HCl buffer, pH 7.0 at the ratio of 1:1. After homogenization, the homogenate was centrifuged at 6000 rpm for 30 min at 4 °C. The supernatant collected after centrifugation was used as the crude digestive enzyme extract and further used for the blue crab α-amylase (BCA) purification.

### 3.3. Protein Concentration and α-Amylase Activity Assays

Protein concentration was estimated according to the method of Bradford [57] using bovine serum albumin as a standard and by measuring the absorbance at 280 nm during the course of enzyme purification.

α-Amylase activity was monitored by quantifying the reducing sugars released during starch hydrolysis using the dinitrosalycilic acid (DNS) method [58]. In brief, the reaction mixture, containing 0.5 mL of the appropriately diluted enzyme and 0.5 mL of 1% (*w/v*) potato starch (Sigma-Aldrich-S4251, Tunisia) in 100 mM Tris-HCl buffer (pH 7.5), was incubated at 50 °C for 10 min. The reaction was terminated by adding 3 mL of the DNS reagent. The mixture was then boiled for 10 min, cooled and absorption was finally measured at 550 nm. One unit of amylase activity was defined as the amount of enzyme that released 1 µmol of the reducing sugar equivalent to glucose per minute under the assay conditions. 

### 3.4. Enzyme Purification Procedure

All purification steps were carried out at 4 °C.

#### 3.4.1. Ultrafiltration

In an attempt to concentrate the amylase activity after extraction, the crude digestive enzyme extract was subjected to ultrafiltration using a stirred ultrafiltration cell (Millipore 8400) equipped with a 10 kDa molecular weight cut-off membrane e (PBGC membrane, Millipore Co., Bedford, MA, USA). The retentate containing the protein of interest was further used as the concentrated crude enzyme preparation. The efficiency of the ultrafiltration process was assessed by retrieving the specific amylolytic activity.

#### 3.4.2. Sephadex G-100 Chromatography

The concentrated crude enzyme extract was loaded onto a Sephadex G-100 gel chromatography column (2.6 cm × 60 cm) previously equilibrated with buffer A (25 mM Tris–HCl, pH 8) containing 0.5% Triton X-100. Fractions of 4 mL were eluted at a flow rate of 30 mL h^−1^ with the same buffer. Protein content and α-amylase activity were determined. The α-amylase-active fractions were collected, pooled and stored at 4 °C for further use.

#### 3.4.3. Anion Exchange Chromatography

Fractions (91–135) showing α-amylase activity were pooled together and applied to a Sepharose Mono Q column (2 cm × 25 cm) previously equilibrated with buffer A (pH 8.0). After washing the column with the same buffer, bound protein elution was performed using a linear gradient of sodium chloride (NaCl) in the concentration range of 0–0.4 M in the equilibrating buffer. Fractions of 3 mL were collected at a flow rate of 75 mL h^−1^ and analyzed for amylase activity and proteins. 

#### 3.4.4. Sodium Dodecyl Sulphate–Polyacrylamide Gel Electrophoresis (SDS-PAGE)

SDS-PAGE was carried out for the determination of purity and molecular weight of the purified BCA α-amylase under reducing conditions as described by Laemmli [59] using 5% (*w/v*) stacking (pH 6.8) and 12% (*w/v*) separating (pH 8.8) gels. Samples were prepared by mixing the purified enzyme at the 4:1 (*v/v*) ratio with distilled water containing 10 mM Tris–HCl, pH 8.0, 2.5% SDS, 10% glycerol, 5% β-mercaptoethanol and 0.002% bromophenol blue. Then, before electrophoresis, samples were heated at 100 °C for 5 min. Finally, the gel was stained with 0.25% Coomassie Brilliant Blue R-250 (Bio-Rad Laboratories, Hercules, California, USA) in 45% ethanol–10% acetic acid and then distained with 5% ethanol–7.5% acetic acid. The enzyme’s molecular weight was estimated using a high molecular weight calibration kit as standard markers from 14.2 to 200 kDa (Amersham Biosciences, Uppsala, Sweden).

#### 3.4.5. Native PAGE and Zymography

The Native PAGE (non-reducing conditions) of the purified BCA was performed according to the procedure of Laemmli [59], except that the sample was not heated and SDS and the reducing agent were left out. Briefly after electrophoresis, the gel was incubated in a renaturation buffer (buffer A) for 60 min at room temperature with gentle shaking. The treated gel was then layered on a thin starch gel containing soluble potato starch (1%) and agar (2%). The agar plates were then incubated for 45 min at 50 °C. After incubation, the polyacrylamide gel was removed, and the agar gel was immersed with iodine solution for 2 min to visualize zones of clearance corresponding to α-amylase activity as transparent bands against a dark blue background.

### 3.5. Biochemical Characterization of the Purified BCA 

#### 3.5.1. Effects of Temperature on Enzyme Activity and Stability

The effect of temperature on BCA activity was studied over the range of 40–90 °C at pH 8.0. In order to monitor thermostability, the purified enzyme was pre-incubated for 60 min at different temperatures ranging from 40 to 70 °C. The effect of substrate (1% starch) addition on BCA thermostability was tested at 60 and 70 °C. Aliquots were withdrawn at desired time intervals and cooled on ice before being assayed to determine residual enzymatic activity under enzyme assay conditions. The non-heated enzyme was used as the control (100%).

#### 3.5.2. Effects of pH on Enzyme Activity and Stability

The effect of pH on BCA was determined by measuring the α-amylase activity over the pH range of 3.0–13.0 at 50 °C. The pH stability was determined by pre-incubating the purified α-amylase in buffers of different pH values in the range of 3.0–13.0 for 1 h at 30 °C. The residual enzymatic activity was determined under the enzyme assay conditions. The following buffer systems were used at 100 mM: glycine–HCl buffer, pH 3.0–4.0; acetate buffer, pH 4.0–6.0; Tris–HCl buffer, pH 7.0–8.0; glycine–NaOH buffer, pH 9.0–11.0; and Na_2_HPO_4_–NaOH buffer, pH 12.0–13.0.

#### 3.5.3. Effects of Metallic Ions on Enzyme Activity

The effects of several metallic ions, assayed at concentrations of 5 mM, were also investigated by adding monovalent (NaCl and KCl) as well as divalent (CaCl_2_, ZnCl_2_, FeCl_2_, HgCl_2_, BaCl_2_, MnCl_2_, MgCl_2_, CoCl_2_, CuCl_2_ and AlCl_2_) ions. Amylolytic activity, measured without any additives, was taken as 100%.

#### 3.5.4. Effects of Inhibitors and Surfactants on Enzyme Stability

The effect of enzyme inhibitors (β-mercaptoethanol and ethylenediaminetetraacetic acid (EDTA)), some non-ionic surfactants (Triton X-100, Tween 20 and Tween 80) and the ionic surfactant (SDS) on enzyme activity was studied. The purified enzyme was pre-incubated at room temperature for 1 h and then the remaining amylolytic activity was measured under enzyme assay conditions. The activity of the enzyme incubated under similar conditions without any additive was taken as 100%.

### 3.6. Kinetic Study and Thin-Layer Chromatographic Analysis

#### 3.6.1. Substrate Specificity Profile

The BCA substrate specificity profile was studied under the standard assay conditions using 1% (*w/v*) starch from various origins, including potato, maize and wheat. Amylose, amylopectin, CMC, α-cyclodextrin, dextrin and chitosan were also used as substrates. The activity for potato starch was defined as 100%.

#### 3.6.2. Kinetic Parameters Determination

The kinetic constants of the purified BCA, including the Michaelis–Menten constant (*K_m_*) and the maximal reaction velocity (*V_max_*), were measured using potato starch as the substrate based on the DNS assay. The reaction was performed by incubating the enzyme in 100 mM Tris–HCl buffer (pH 7.5) supplemented with varying concentrations of potato starch (0.5–20 mg mL^−1^). The reaction was performed at 50 °C for 10 min. Each assay was carried out in triplicate, and kinetic parameters were estimated using Lineweaver–Burk plots. 

#### 3.6.3. TLC Analysis of Starch and Maltooligosaccharides Hydrolysis Products 

In an attempt to analyze BCA reaction products, potato starch and various maltooligosaccharides (G2–G8) were hydrolyzed by the purified BCA α-amylase, using 1% (*w/v*) substrate in 100 mM Tris–HCl buffer (pH 7.5) at 50 °C. The hydrolysis products withdrawn at different incubation times were subjected to thin-layer chromatography (TLC) with silica gel 60 (20 cm × 20 cm, Merck, Germany) in a solvent system consisting of chloroform/acetic acid/water (60:70:10, *v/v/v*). The spots were finally visualized by spraying TLC plates with sulfuric acid/ethanol (5:95, *v/v*) followed by heating at 120 °C for 10 min in an oven until spots appeared. Starch and G2, G3, G5, G6, G7 and G8 were used as standards.

### 3.7. Storage Stability

The enzyme was stored for 90 days and 1 year at 4 °C in 25 mM Tris–HCl buffer, pH 7.5, to assess the enzyme’s stability. Residual activity was measured at regular time intervals. Initial activity measured at the first day of storage was regarded as 100%.

### 3.8. Application of Purified BCA for the Improvement of Antioxidant Potential of Oat

Oat grains were ground to powder and screened in a 1 mm sieve immediately prior to analysis and 0.4 g of the resulting powder was mixed with 9.5 mL of the Tris–HCl buffer (pH 7.5). Then, 500 µL of the purified α-amylase was added. Hydrolysis was carried out in a water bath at the temperature of 50 °C for 1 h. The reaction was stopped by increasing temperature of the mixture to 100 °C for 5 min. The control was prepared with the addition of 500 µL Tris–HCl buffer instead of the enzyme. The above reaction mixture was centrifuged and the clear supernatant was used to quantify the total soluble phenolic content (TSPC) of the control and BCA-treated oats according to the method of Slinkard and Singleton [60]. DPPH scavenging activity was estimated for the determination of antioxidant potential of samples according to the method of Brand–Williams et al. [61]. Gallic acid was used as the positive control.

### 3.9. Statistical Analysis

All determinations were performed at least in three independent replicates. Results were expressed as the mean of the replicate determinations and standard deviation (SD) (mean ± SD). The results were considered statistically significant for p-values of less than or equal to 0.05.

## 4. Conclusions

The marine world represents a largely untapped reservoir of bioactive molecules that can be applied in food biotechnology. Considering the expensive production of enzymes from microbial sources, marine-derived ones are highly preferred, especially when considering visceral byproducts produced as a result of fish processing as a source of marine enzymes. In the present study, a novel digestive α-amylase (BCA) extracted from blue crab viscera was purified and characterized. After the final purification step, the enzyme was purified to homogeneity (424.02-fold) with the specific activity of 1390.8 U mg^−1^ and 28.8% recovery. The molecular weight of the purified BCA was estimated to be 45 kDa, as shown by the SDS-PAGE analysis. The enzyme’s kinetic parameters *K_m_* and *V_max_* values were 7.5 ± 0.25 mg mL^−1^ and 2000 ± 23 μmol min^−1^ mg^−1^, respectively. The enzyme possesses desirable biotechnological features, such as the optimal temperature of 50 °C, interesting thermal stability, which was enhanced in the presence of starch, high specific activity and broad pH range stability. BCA hydrolyzed various carbohydrates and produced maltose, maltotriose and maltotetraose as the major end products of starch hydrolysis, supporting its potential application in the production of maltodextrin-rich syrups free of glucose. In addition, the purified enzyme was successfully utilized for the improvement of antioxidant potential of oat flour, which could be extended to other cereals. Overall, the purified enzyme from blue crab viscera may be suitable for application in biotechnological and industrial processing owing to its interesting features.

## Figures and Tables

**Table 1:** Summary of purification of the digestive α-amylase (BCA) from blue crab viscera.

Purification Steps	Total Activity (U)	Total Protein (mg)	Specific Activity (U mg^−1^)	Recovery (%)	Purification Fold
Crude visceral extract	3000.00	913.70	3.28	100.00	1.00
Ultrafiltration (10 kDa)	2550.00	165.00	15.45	85.00	4.71
Sephadex G-100	1470.00	14.40	102.08	49.00	31.12
Mono Q Sepharose	834.50	0.60	1390.80	27.80	424.02

All operations were carried out at 4 °C.

**Table 2:** Effects of various metal ions (5 mM) on BCA activity.

Metal Ions (5 mM)	Relative Activity (%)
None	100.00
Ca^2+^	97.00 ± 1.40
Fe^2+^	17.40 ± 0.84
Mg^2+^	109.70 ± 0.67
Ba^2+^	118.50 ± 2.12
Hg^2+^	0
Zn^2+^	0
Mn^2+^	6.20 ± 0.42
Co^2+^	18.75 ± 0.35
Cu^2+^	8.40 ± 0.56
Al^2+^	91.30 ± 0.98
K^+^	95.50 ± 0.61
Na^+^	100.00

The activity of the α-amylase was determined by incubating the enzyme in the presence of various metal ions (5 mM) for 10 min at 50 °C and pH 7.5.

**Table 3:** Effects of surfactants and enzyme inhibitors on BCA stability.

Chemical Reagents	Concentration	Remaining Activity (%)
None	---	100.00
Surfactants		
Tween 20	5% (*v/v*)	71.13 ± 1.49
Tween 80	5% (*v/v*)	71.00 ± 0.42
Triton X-100	5% (*v/v*)	80.78 ± 2.52
SDS	1% (*w/v*)	26.20 ± 1.54
	2% (*w/v*)	17.55 ± 1.48
Inhibitors		
β-mercaptoethanol	5 mM	93.90 ± 2.68
EDTA	5 mM	92.89 ± 0.84

Additives were pre-incubated with the purified enzyme for 1 h at room temperature. The remaining amylolytic activity was measured under enzyme assay conditions. Enzyme activity measured in the absence of any additives was taken as 100%.

**Table 4:** Relative activities of purified BCA on various substrates (1%).

Substrates	Relative Activity (%)
Potato starch	100.00
Maize starch	83.40 ± 1.55
Wheat starch	96.20 ± 1.05
Amylose	17.80 ± 2.25
Amylopectin	71.13 ± 0.74
CMC	0
α-Cyclodextrin	0
Dextrin	0
Chitosan	0

**Table 5:** Improvement of antioxidant potential of oat flour by purified BCA.

Sample	α-Amylase Activity (Units) or Gallic Acid Concentration (µg mL^−1^)	DPPH Scavenging Activity (% of Inhibition)	Total Soluble Phenolic Compounds (TSPC) (Gallic Acid Equivalent in µg g^−1^ Oat)
Native oat (4%)	0	38.40 ± 1.15	23.00 ± 2.50
BCA-treated oat	10.00	67.53 ± 2.05	75.00 ± 4.25
Gallic acid *	—	50.00	—

* Positive control of the DPPH scavenging activity (4.69 µg/mL).

## Data Availability

The data that support the findings of this study are available from the corresponding author upon reasonable request.
